# Obituary

**Published:** 1896-01

**Authors:** 


### Obituary.

Died on December 23d, 1895, at her home, in Nashville, Tennessee, Mrs. Sarah A. Morgan, wife of Dr. W. H. Morgan. A local paper says: "Dr. W. H. Morgan and his wife have long been two of the best known, and most highly esteemed citizens of Nashville, and the bereavement of the one by the death of the other will strike a sympathetic cord in the hearts of all their multitudes of friends."

Dr. Morgan is known, not only in his own city and State, but throughout the United States, and every where has hosts of friends, whose hearts will be made sad by this great bereavement that has come upon him. He will have the sincere sympathy of all.

May the Dr. and all the members of his family have the support and strength they now so much need, and which can only come from our Heavenly Father, who doeth all things well.

